# The appropriate number of ELNs for lymph node negative breast cancer patients underwent MRM: a population-based study

**DOI:** 10.18632/oncotarget.20052

**Published:** 2017-08-07

**Authors:** Huiying Chi, Chenyue Zhang, Haiyong Wang, Zhehai Wang

**Affiliations:** ^1^ Shanghai Geriatrics Institute of Traditional Chinese Medicine, Shanghai 200032, China; ^2^ Department of Integrative Oncology, Fudan University Shanghai Cancer Center, Shanghai 200032, China; ^3^ Department of internal Medicine-Oncology, Shandong Cancer Hospital and Institute, Shandong Cancer Hospital affiliated to Shandong University, Shandong Academy of Medical Sciences, Jinan 250117, China

**Keywords:** breast cancer, mastectomy, ELNs, lymph nodal negative, X-tile

## Abstract

Whether number of examed lymph nodes (ELNs) would bring survival benefit for patients with negative lymph nodes after modified radical mastectomy (MRM) is uncertain. In our study, using the Surveillance Epidemiology and End Results (SEER) database between 2004 and 2009, we screened the appropriate patients with negative lymph nodes underwent MRM. The Cox proportional hazard analysis was used to determine the effect of number of ELNs on cancer specific survival (CSS). The results showed that the number of ELNs was not an independent prognostic factor on CSS (*P* = 0.940). Then the X-tile mode was used to determine the appropriate threshold for ELNs count. The results showed that 9 was the appropriate cut-off point. Next, the log-rank χ^2^ test was used to analyze the CSS based on different subgroup variables. The results showed that some subgroup variables including age < 50/ ≥ 50, grade I/III, AJCC T1/T2, ER positive/negative and PR positive/negative ,demonstrated significant CSS benefits among the patients with the number of ELNs ≤ 9 (all, *P* < 0.05). However, three subgroup variables including grade II, AJCC T3 and AJCC T4, the patients with the number of ELNs ≤ 9 did not bring significant CSS benefits (all, *P* > 0.1). In conclusion, our study demonstrated that the number of ELNs was not an independent prognostic factor on CSS, and 9 can be selected as the appropriate cut-off point of ELNs for patients with negative lymph nodes who underwent MRM.

## INTRODUCTION

Breast cancer is the most frequently diagnosed cancer and the leading cause of cancer death among females worldwide, with an estimated 1.7 million incidence and 521,900 mortalities in 2012 [1]. Breast cancer alone accounts for 25% of all cancers and 15% of all cancer-related deaths among females [1].

Axillary lymph node status has been widely acknowledged as an important prognostic factor for breast cancer patients underwent MRM [2–4]. It is well known that the number of positive lymph nodes (PLNs) is strongly associated with the tumor node metastasis (TNM) staging system and would determine the strategies of adjuvant therapy [5–8]. National Comprehensive Cancer Network (NCCN) guidelines and many studies have recommended post-mastectomy radiation therapy as a candidate therapy for breast cancer patients based on the number of PLNs [9–11]. Interestingly, other expression mode of lymph node status including the ratio of positive node defined as the number of PLNs divided by the total number of ELNs, the number of negative lymph nodes (NLNs) defined as the total number of ELNs by axillary dissection minus the number of PLNs, have also been introduced and even showed more significance in predicting the prognosis of breast cancer patients [12–15]. Importantly, the expression mode of lymph node is strongly dependent on the total number of ELNs. Theoretically, more number of ELNs may provide more accurate information on TNM classification and breast cancer prognosis by increasing the probability of PLNs. However, for the breast cancer patients with negative lymph nodes after MRM, whether more number of ELNs would bring survival benefit is uncertain. In addition, the appropriate number of ELNs should also be determined for these breast cancer patients.

To address these unknown issues of clinical significance, using the Surveillance, Epidemiology, and End Results (SEER)-registered database, we analyzed the association between the number of ELNs and prognosis of breast cancer patients. Importantly, we used an X-tile mode to determine an appropriate threshold for ELNs count.

## RESULTS

### Patient demographics

There were 15633 female breast cancer patients reported in the SEER database from 2004 to 2009. These patients were followed up for a consecutive 119 months. The clinical characteristics of all the patients were summarized in Table 1. The number of ELNs ranged from 1 to 81. Most patients were white-raced (79.1%) and diagnosed at the age of more than 50-year-old (74.1%). the proportion of patients with Grade I, II and III was 19.8%, 41.0% and 39.2%, respectively. Interestingly, most of the patients were diagnosed at the AJCC stage T1–2 (91.3%). In addition, most patients were not received radiation (87.7%), and the positive rates of ER and PR status were 72.1% and 61.9%, respectively. The detailed characteristics were shown in Table 1.

**Table 1:** Characteristics of lymph node negative breast cancer patients underwent modified radical mastectomy from SEER Database from 2004–2009

Variables	Number (%)
Total	15633 (100)
Number of ELN	1–81
**Age**	
< 50	4042 (25.9)
≥ 50	11591 (74.1)
**Race**	
White	12365 (79.1)
Black	1692 (10.8)
Others	1576 (10.1)
**Grade**	
I	3103 (19.8)
II	6403 (41.0)
III	6127 (39.2)
**T stage**	
T1	8769 (56.1)
T2	5496 (35.2)
T3	870 (5.6)
T4	498 (3.2)
**Radiation**	
Yes	1922 (12.3)
No	13710 (87.7)
**ER status**	
Positive	11271 (72.1)
Negative	4362 (27.9)
**PR status**	
Positive	9677 (61.9)
Negative	5956 (38.1)

### Number of ELNs and CSS

**Figure 1:**
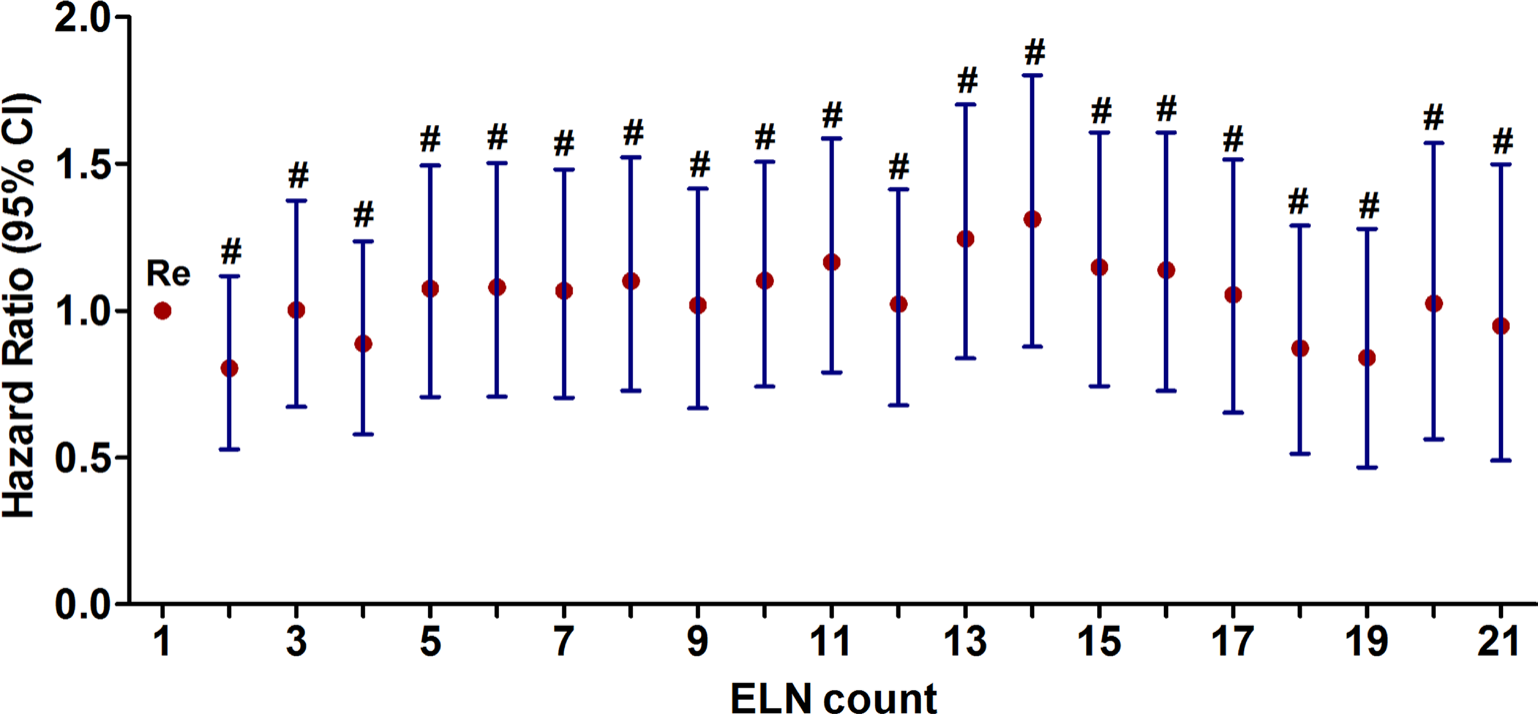
The correlation between the number of ELNs as a categorical variable and CSS adjusted for other variables using the Cox proportional hazard analysis (^#^*P* > 0.05).

After controlling for other prognostic factors including age, race, grade, AJCC T stage, radiation, ER status and PR status, the Cox proportional hazard analysis was used to analyze the correlation between the number of ELNs (as a continuous variable) and the CSS. Interestingly, our results showed that the number of ELNs was not an independent prognostic factor on CSS (hazard ratio (HR):1.000; confidence interval (CI) 95%: 0.992–1.008; *P* = 0.940) (Table 2). Next, we regarded the number of ELNs = 1 as the reference, the Cox proportional hazard analysis was also used to analyze the correlation between the number of ELNs (as a categorical variable) and the CSS. Our results showed that the increased number of ELNs did not bring benefit for CSS (all, *P* > 0.1) (Figure 1).

**Table 2:** Univariate and multivariate Cox regression analysis were used to evaluate the influence of different variables on CSS for lymph node negative breast cancer patients underwent modified radical mastectomy

Variables	Univariate analysis		Multivariate analysis	
	Wald χ^2^	*P*	HR (95%CI)	*P*
**Number of ELN**	**22.651**	**< 0.001**	**1.000 (0.992–1.008)**	**0.940**
**Age**	4.779	0.029		< 0.001
< 50			reference	
≥ 50			1.426 (1.251–1.626)	< 0.001
**Race**	0.128	0.720	Not included	
White				
Black				
Others				
**Grade**	324.633	< 0.001		< 0.001
I			reference	
II			2.026 (1.551–2.647)	< 0.001
III			3.165 (2.417–4.144)	< 0.001
**T stage**	707.957	< 0.001		< 0.001
T1			reference	
T2			2.397 (2.085–2.755)	< 0.001
T3			3.825 (3.097–4.725)	< 0.001
T4			6.512 (5.273–8.042)	< 0.001
**Radiation**	127.075	< 0.001		0.998
Yes			reference	
No			1.000 (0.856–1.169)	0.998
**ER status**	270.691	< 0.001		0.020
Positive			reference	
Negative			1.224 (1.033–1.452)	0.020
**PR status**	240.919	< 0.001		0.001
Positive			reference	
Negative			1.323 (1.119–1.565)	0.001

### Determine the appropriate cut-off point of ELNs count

**Figure 2:**
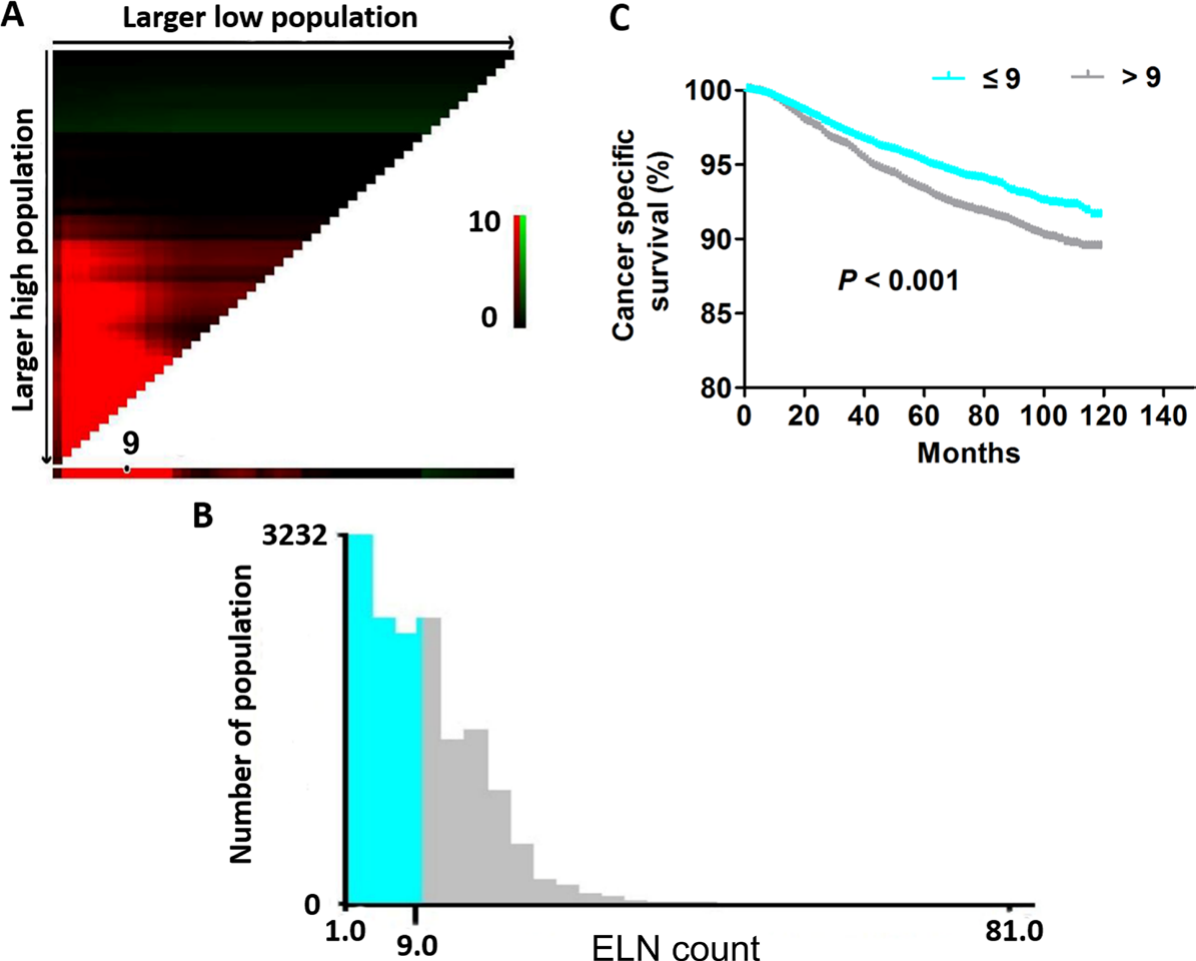
The appropriate cut-off point of ELNs count using the X-tile model (**A**) X-tile plots of matched number of ELNs. (**B**) The optimal cut-off point highlighted by the gray and green panel. (**C**) The CSS curves between the patients with the number of ELNs > 9 and ≤ 9 (*P* < 0.001).

Since the number of ELNs was not an independent prognostic factor on CSS, it is necessary to identify the optimal cut-off count of lymph nodes for patients with negative lymph nodes. The X-tile mode was used to determine the appropriate cut-off point of ELNs count. Results showed 9 was the appropriate cut-off point (Figure 2A). The Kaplan-Meier analyses were used to generate the survival curves in X-tile mode, and the Log Rank test was applied to compare the prognosis between the patients with the number of ELNs > 9 and the number of ELNs ≤ 9. The results showed that the patients with the number of ELNs ≤ 9 had a better CSS rate (*P* < 0.001) (Figure 2B and 2C).

### Validate the cut-off point of ELNs count

In order to further verify the feasibility of the 9 as the appropriate cut-off point for ELNs count, we further analyzed individual number of ELNs from 1 to 15 using univariate log-rank test. The 5-year CSS with different numbers of ELNs were calculated, respectively. The results showed that the patients with more than nine ELNs count had a maximum log-rank χ^2^ value in CSS (χ^2^ = 25.59, *P* < 0.001) (Table 3). Therefore, 9 can be selected as the appropriate cut-off point for these patients. The 5-year CSS rate of the patients with the number of more than nine ELNs count was 93.3% (Table 3). The 5-year CSS rate of the patients with the number of nine and fewer than nine ELNs count was 95.2% (Table 3). Therefore, the cut-off 9 was validated as the appropriate threshold.

**Table 3:** The log rank χ^2^ test to evaluate the influence of difference cut-off point on CSS for lymph node negative breast cancer patients underwent modified radical mastectomy

Cutoff	Number	5-year CSS (%)	Log rank χ^2^	*P*
≤ 1	943	94.8	2.25	0.134
> 1	14690	94.2		
≤ 2	2116	96.1	13.95	< 0.001
> 2	13517	94.0		
≤ 3	3232	95.8	17.90	< 0.001
> 3	12401	93.8		
≤ 4	4162	95.9	24.61	< 0.001
> 4	11471	93.7		
≤ 5	4944	95.7	23.56	< 0.001
> 5	10689	93.6		
≤ 6	5729	95.5	24.01	< 0.001
> 6	9904	93.5		
≤ 7	6531	95.4	23.86	< 0.001
> 7	9102	93.4		
≤ 8	7344	95.2	24.89	< 0.001
> 8	8289	93.4		
≤ 9	8093	95.2	25.59	< 0.001
> 9	7540	93.3		
≤ 10	8955	95.0	22.18	< 0.001
> 10	6678	93.2		
≤ 11	9771	94.9	14.23	< 0.001
> 11	5862	93.1		
≤ 12	10589	94.8	15.58	< 0.001
> 12	5044	93.1		
≤ 13	11348	94.7	9.96	0.002
> 13	4285	93.0		
≤ 14	12022	94.6	4.92	0.027
> 14	3611	93.2		
≤ 15	12602	94.5	3.00	0.083
> 15	3031	93.3		

### Subgroup analysis for CSS based on the appropriate threshold

**Figure 3:**
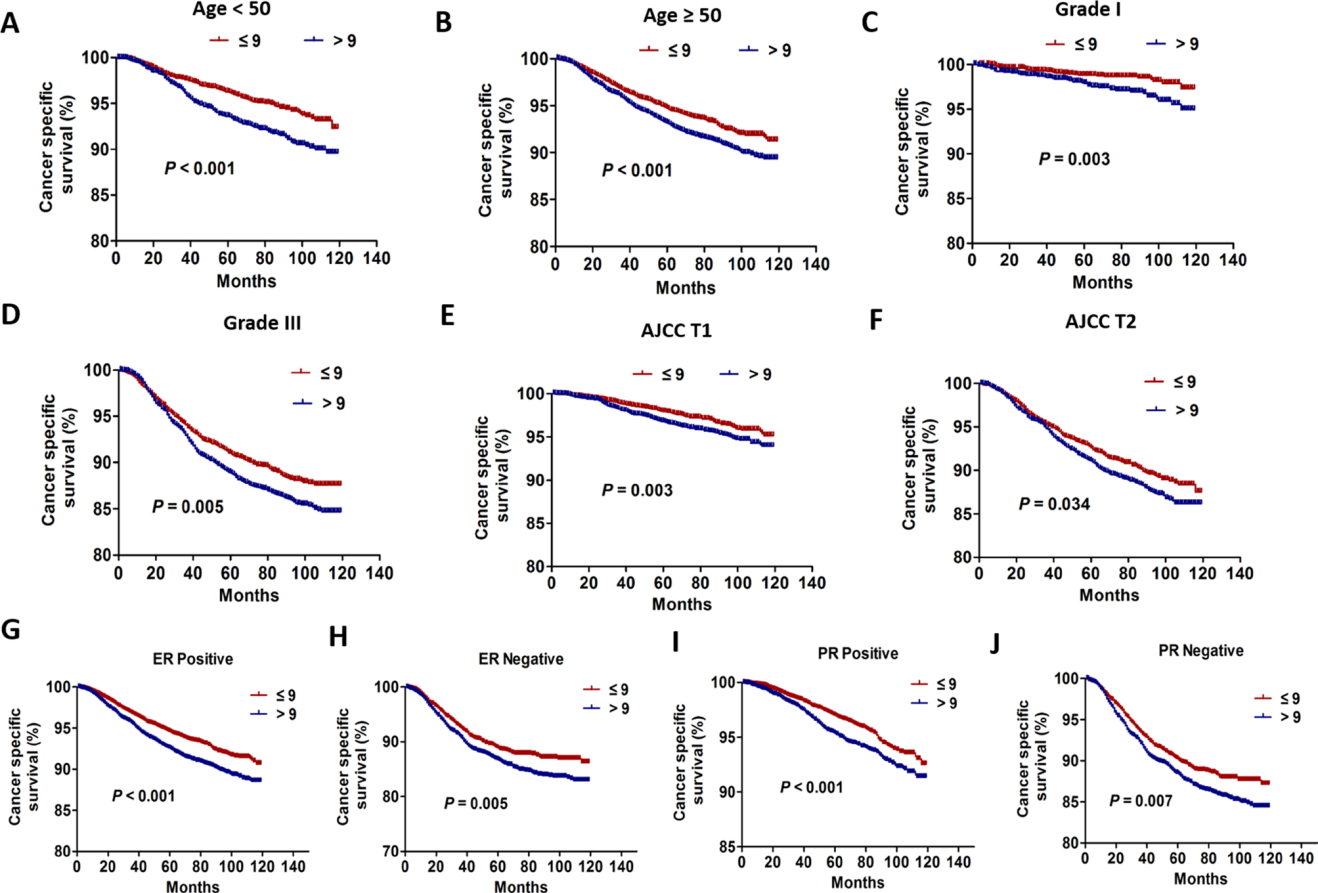
The CSS curves between the patients with the number of ELNs > 9 and ≤ 9 according to different variables (**A**) The CSS curves of patients aged < 50 (*P* < 0.001). (**B**) The CSS curves of patients aged ≥ 50 (*P* < 0.001). (**C**) The CSS curves of patients with grade I (*P* = 0.003). (**D**) The CSS curves of patients with grade III (*P* = 0.005). (**E**) The CSS curves of patients with AJCC T1 (*P* = 0.003). (**F**) The CSS curves of patients with AJCC T2 (*P* = 0.034). (**G**) The CSS curves of patients with ER positive (*P* < 0.001). (**H**)The CSS curves of patients with ER negative (*P* = 0.005). (**I**) The CSS curves of patients with PR positive (*P* < 0.001). (**J**) The CSS curves of patients with PR negative (*P* = 0.007).

The Cox proportional hazard analysis has shown that variables including age, grade, and AJCC T stage ER and PR status were all independent prognostic factors on CSS (Table 2). Next, subgroup analysis using the log-rank χ^2^ test was conducted and the survival curves were formed based on the appropriate threshold. The results showed that the subgroup variables including age < 50, age ≥ 50, grade I, grade III, AJCC T1,AJCC T2, ER/PR positive and ER/PR negative, the number of ELNs ≤ 9 all demonstrated significant CSS benefits (age < 50: log-rank χ^2^: 13.76, *P* < 0.001; age ≥ 50: log-rank χ^2^: 13.24, *P* < 0.001; grade I: log-rank χ^2^: 8.79, *P* = 0.003; grade III: log-rank χ^2^: 8.02, *P* = 0.005; AJCC T1: log-rank χ^2^: 8.90, *P* = 0.003; AJCC T2: log-rank χ^2^: 4.51, *P* = 0.034; ER positive: log-rank χ^2^: 26.78, *P* < 0.001; ER negative: log-rank χ^2^: 7.88, *P* = 0.005; PR positive: log-rank χ^2^: 12.00, *P* < 0.001; PR negative: log-rank χ^2^: 7.19, *P* = 0.007 ) (Figure 3A–3J). However, three subgroup variables including grade II, AJCC T3 and AJCC T4, the number of ELNs ≤ 9 didn’t show significant CSS benefits (grade II: log-rank χ^2^: 0.96; *P* = 0.329; AJCC T3: log-rank χ^2^: 0.40; *P* = 0.529; AJCC T4: log-rank χ^2^: 1.61; *P* = 0.204) (Supplementary Figure 1A–1C).

## DISCUSSION

Theoretically, more number of ELNs may provide accurate information on TNM classification and prognosis in breast cancer patients by increasing the probability of PLNs. In fact, some studies have demonstrated that a strong correlation between the number of ELNs and survival benefit of patients as described [16, 17]. However, these studies are mainly based on the patients with positive lymph nodes. For the breast cancer patients with negative lymph nodes after MRM, our results demonstrated that the increased number of ELNs was not an independent prognostic factor on CSS and did not bring CSS benefit, which were also supported by other studies [18–20]. For instance, moorman et al demonstrated that the number of ELNs was found to be associated with neither 5-year nor long-term survival; the proportion of women dying from breast cancer was the same in both groups (those patients with > or = 20 lymph nodes examined vs. those in whom < 20 lymph nodes were examined) [18]. Interestingly, Camp et al. even find a worse 5-year survival for patients with tumor free lymph nodes more than 20 compared with patients with fewer than 20 tumor free lymph nodes [20]. However, some studies have inconsistent conclusion. For example, a study by van der wal et al showed that node-negative patients with < 14 lymph nodes removed had a 10 year survival of 79% compared with 89% in patients with > or = 14 lymph nodes removed [21]. Another study by Krag et al showed that even when all regional lymph nodes are pathologically negative, the number of nodes removed is associated with survival; In the group of negative-node breast cancer patients, the increased number of removed nodes is associated with improved survival; The hazard rate for death in the node-negative group was roughly 5% less for each additional five nodes removed [17]. The reason for the difference may be attributed to the fact that only patients undergoing MRM were enrolled in our study.

We have demonstrated that increasing number of ELNs did not bring any survival benefit. Therefore, it is important to determine the appropriate number of ELNs. We therefore used X-tile model to determine the optimal cutoff value. X-tile plots provide a single, global assessment of every possible way of dividing a population into low-, medium-, and high- level marker expression [22]. In addition, X-tile can produce corrected *p* values using several Monte Carlo simulations: Cross-Validation takes our dataset, randomly splits it into two halves, finds the optimal cut-point of one half, and then divides the other half according to this cut-point. Then, it finds the optimal cut-point of the second half and similarly divides the first. [22]. The X-tile plots have been applied by other study to determine the appropriate cut-off value [23]. Our results demonstrated that 9 can be selected as the appropriate cut-off point for these patients. In addition, using the minimum *p* value method used in others references [24, 25], we further verify the feasibility of 9 as appropriate cut-off point for ELNs count. In fact, a study from Danish Breast Cancer Cooperative Group in 1992 has demonstrated that the number of lymph nodes removed should be at least up to 10 to exclude misclassification of node-positive patients as node negative [16]. However, to the best of our knowledge, we first developed a cut-off for ELNs among a huge group of breast cancer patients. Our findings might give some advice to specialists in surgery to determine the extent of lymph node dissection.

Importantly, in our study, the log-rank χ^2^ test was applied and subgroup analysis was conducted. It is worth noting that for the subgroup patients with AJCC T3 and T4, the CSS difference was not found between the patients with ≤ 9 ELNs and > 9 ELNs. In fact, some studies have demonstrated that in breast cancer patients, there is a strong correlation between tumor size and the risk of axillary lymph nodes involvement [26–28]. For the subgroup patients with AJCC T3 and T4, it was not appropriate to regard nine as the cut-off point of ELNs. Therefore, further studies are needed to determine the number of ELNs in combination with other prognostic factors among breast cancer patients. The ELNs rate should be further assessed, especially for the high-risk patients.

Undeniably, this study also has several limitations. Firstly, as the non-randomized study, the intrinsic defects exist in any retrospective study despite a relatively larger sample size. Secondly, some important prognosis factors such as HER-2, Ki67 status were not included in the present study.

In conclusion, our study demonstrated that the number of ELNs was not an independent prognostic factor on CSS. Importantly, our results demonstrated that 9 can be selected as the appropriate cut-off point of ELNs for patients with negative nodes who underwent modified radical mastectomy. Of course, further randomized controlled studies and sufficient subgroup analyses are warranted to validate our conclusion.

## MATERIALS AND METHODS

### Patient selection

The SEER Cancer Statistics Review (http://seer.cancer.gov/data/citation.html) is published annually by the Data Analysis and Interpretation Branch of the National Cancer Institute, MD, USA. A total of 18 population-based cancer registries in the United States were included in the current SEER database [29]. The SEER*Stat software (SEER*Stat 8.3.2) was used to identify the appropriate patients. Using this software, we screened female breast cancer patients whose histological type are limited to ductal and lobular neoplasms between January 1, 2004 and December 31, 2009. The included patients should meet the following criteria: the diagnosis was confirmed microscopically, they should be female with the confirmed age, active follow-up and only one primary tumor. In addition, the patients should be those who have received modified radical mastectomy, with no positive lymph nodes removed. Patients with benign or borderline tumors were excluded. And patients lacking information on age, race, grade, AJCC T stage, ER status and PR status, the number of examed lymph nodes, cause of death, survival months were also excluded.

### Ethics statement

This study was mainly based on the SEER database and was conducted in compliance with the Helsinki Declaration. We obtained permission to access the files of SEER program research data and the reference number is 11304-Nov 2015. The informed consent was not required because personal identifying information was not involved. This study was approved by the ethics committee of the Shandong Cancer Hospital affiliated to Shandong University.

### Statistical analysis

For all the patients, the following variables were analyzed: Age, race, grade, AJCC T stage, radiation, ER status and PR status, and the number of ELNs. In addition, CSS were regarded as the primary endpoint of this study and extracted from the SEER database. CSS is a survival measure representing survival of a specified cancer of death in the absence of other causes of death. The Cox proportional hazard analysis was used to determine the effect of number of ELNs on CSS adjusted for other significant prognostic factors. The number of ELNs is actually the number of lymph nodes removed after operation. The X-tile mode was used to determine the appropriate threshold for ELNs count. Then based on the appropriate threshold, the log-rank χ2 test was also performed to analyze the CSS based on different subgroup variables. In addition, the appropriate threshold was validated by using Log rank χ^2^ test based on different cut-off values. All statistical tests were two-sided, and a *P* < 0.05 was considered statistically significant. The statistical software SPSS 18.0 (SPSS, Chicago, IL, USA) was used for all data analyses.

## SUPPLEMENTARY MATERIALS FIGURE



## Abbreviations

*ELNs*: *examed lymph nodes*
*PLNs*: *positive lymph nodes*
*CSS*: *cancer specific survival*
*SEER*: *surveillance epidemiology and end results*
*NLNs*: *negative lymph nodes*
