# Supplementary material for: The appropriate number of ELNs for lymph node negative breast cancer patients underwent MRM: a population-based study
Source: Oncotarget. 2017 Aug 7;8(39):65668–76. doi: 10.18632/oncotarget.20052 (PMC5630362; doi:10.18632/oncotarget.20052)
Supplement: Supplementary file 1 [file oncotarget-08-65668-s001.pdf]

The appropriate number of ELNs for lymph node negative breast cancer patients underwent MRM: a population-based study

SUPPLEMENTARY MATERIALS

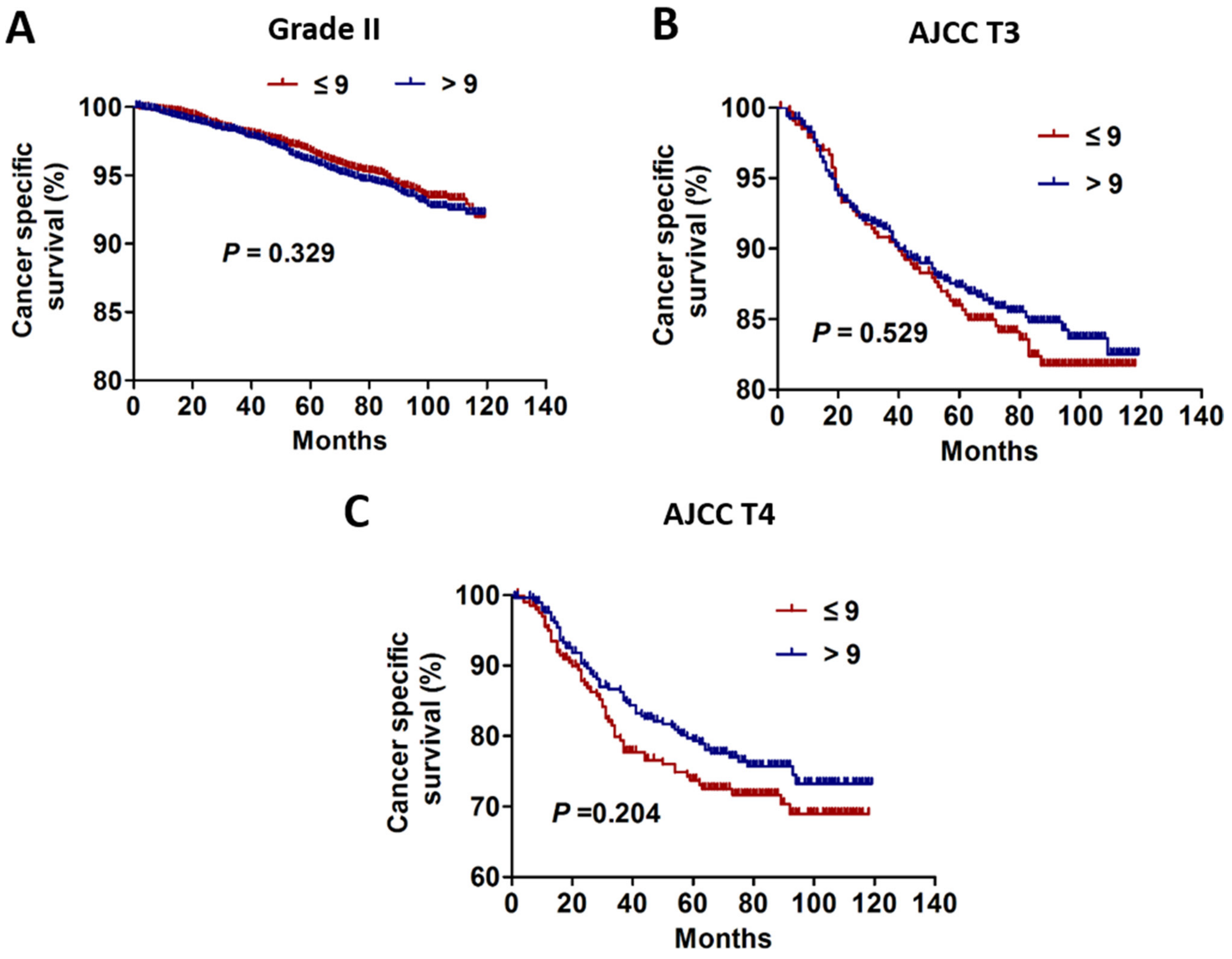

Supplementary Figure 1: The CSS curves between the patients with the number of ELNs > 9 and ≤ 9 according to different variables. (**A**) The CSS curves of patients with grade II (P = 0.329). (**B**) The CSS curves of patients with AJCC T3 (P = 0.529). (**C**) The CSS curves of patients with AJCC T4 (P = 0.204).
